# Prognosis of Atrial Fibrillation Patients Undergoing PCI According to Anticoagulants and Antiplatelet Agents

**DOI:** 10.3390/jcm10153370

**Published:** 2021-07-29

**Authors:** Gwang-Seok Yoon, Sun-Hwa Kim, Si-Hyuck Kang, Chang-Hwan Yoon, Young-Seok Cho, Tae-Jin Youn, In-Ho Chae

**Affiliations:** 1 Cardiovascular Center, Seoul National University Bundang Hospital, Seongnam-si 13620, Korea; shiningstonegs@gmail.com (G.-S.Y.); sunsink02@gmail.com (S.-H.K.); eandp303@snu.ac.kr (S.-H.K.); flammeus@snubh.org (Y.-S.C.); ytjmd@snubh.org (T.-J.Y.); ihchae@snubh.org (I.-H.C.); 2 Department of Internal Medicine, Seoul National University, Seoul 03087, Korea

**Keywords:** atrial fibrillation, percutaneous coronary intervention, anticoagulant, antiplatelet

## Abstract

There are limited data evaluating conformation of antithrombotic therapy usage to the guideline recommendations. We investigated clinical trends and prognoses of patients with atrial fibrillation (AF) according to anticoagulants and antiplatelet agents beyond 1 year after percutaneous coronary intervention (PCI). We analyzed the records of patients with AF who underwent PCI using the Korean National Health Insurance Service database. The primary endpoint was a composite of major adverse cardiac events (MACE). The safety outcome was bleeding complications. Of 4193 participants, 81.6% received antiplatelet therapy, whereas 27.3% had oral anticoagulant (OAC)-based therapy at 18 months after PCI. The dominant therapy was dual antiplatelet therapy (37.2%), and only 3.3% of participants had OAC monotherapy. At the 1-year follow-up, the incidence of MACE was significantly lower among those receiving a combination of OAC and single antiplatelet therapy (SAPT) than among those receiving OAC monotherapy (4.78% vs. 9.42%, *p* = 0.017). Bleeding complication events (5.01% vs. 5.80%, *p* = 0.587) did not differ between the groups. In clinical practice, most patients with AF who underwent PCI continued to receive antiplatelet agents beyond 1-year post-PCI. OAC with SAPT seemed to be more effective than OAC monotherapy, without a difference in safety.

## 1. Introduction

Long-term oral anticoagulant therapy (OAC) is indicated in approximately 5–7% of patients with coronary artery disease (CAD) who are undergoing percutaneous coronary intervention (PCI) [1]. CAD is estimated to occur in 20–40% of patients with atrial fibrillation (AF) [2,3]. Patients with AF require OACs to reduce the risk of thromboembolic events [4], whereas antiplatelet therapy is essential to prevent thrombotic events, including stent thrombosis, in patients with CAD [5]. Therefore, a combination of antithrombotic therapy including OAC and antiplatelet therapy is recommended for patients with AF undergoing PCI and stent implantation [5,6,7]. However, there are concerns about the clinical benefit of the aforementioned intensive combination antithrombotic therapy, as it increases the risk of fatal bleeding, which would offset the benefit of reducing ischemic risk [8,9,10,11]. A balance between these therapies should be maintained to avoid thromboembolic events, such as myocardial infarction (MI) or stroke and bleeding events, in patients with AF.

Current guidelines recommend triple therapy, which includes an OAC, along with aspirin and a P2Y12 inhibitor, for an as-short-as-possible period, for the immediate antithrombotic treatment of patients with AF who have undergone PCI, and for whom the ischemic risk outweighs the risk of bleeding. In selected patients, such treatment is followed by combination therapy, which includes an OAC plus a P2Y12 inhibitor for up to 12 months. After 12 months of combination therapy, or in patients with AF and stable CAD not requiring intervention, current guidelines recommend monotherapy with an OAC [5,6,7].

Limited data are available regarding the current antithrombotic therapy and outcomes of patients with AF beyond 1 year after PCI. Therefore, we investigated the clinical trends and the prognosis of patients with AF beyond 12 months after PCI according to combinations of anticoagulants and antiplatelet agents by using the Korean National Health Insurance database.

## 2. Materials and Methods

### 2.1. Data Source

The National Health Insurance Services (NHIS) claims database was used in this study. The NHIS is an obligatory universal health insurance system covering up to 97% of the entire Korean population. The NHIS claims database contains sociodemographic information, information regarding the use of inpatient and outpatient health care, pharmacy dispensing claims, and mortality data. This study was approved by the Seoul National University Bundang Hospital Institutional Review Board (B-1902-522-001). Informed consent was waived because of the retrospective nature of the study and the use of anonymous clinical data for analysis.

### 2.2. Definitions

The definition of AF was determined using the International Classification of Disease 10th Revision Clinical Modification codes (I48.0-I48.4 and I48.9). To ensure diagnostic accuracy, patients in whom AF was recorded as the discharge diagnosis and/or AF had been confirmed more than twice in the outpatient clinic were defined as having AF. The procedure codes for PCI (M6561, M6562, M6563, and M6564) were used to identify patients who had undergone PCI. The definitions of co-morbidities were based on diagnostic codes and are summarized in Supplementary Table S1. CHA_2_DS_2_-VASc scores were calculated for each patient to assess the individual stroke risk.

### 2.3. Study Population

**Figure 1:**
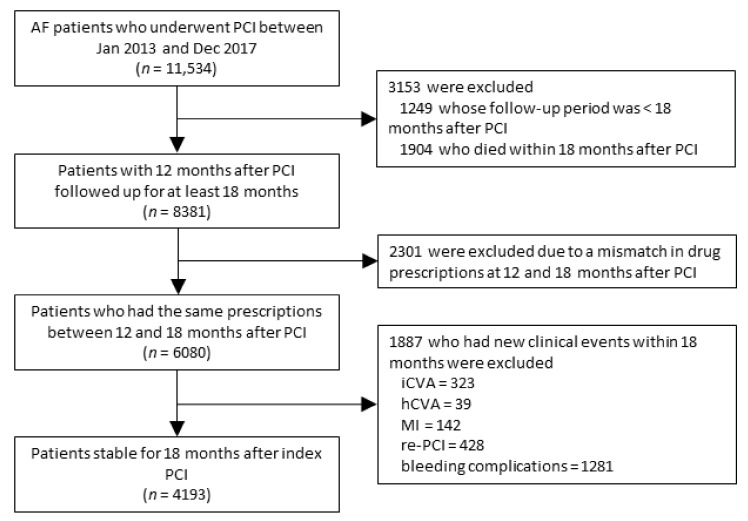
Study enrollment flow. AF, atrial fibrillation; hCVA, hemorrhagic cerebrovascular accident; iCVA, ischemic cerebrovascular accident; MI, myocardial infarction; PCI, percutaneous coronary intervention.

We identified patients diagnosed with AF who underwent PCI between 1 January 2013, and 31 December 2017. We initially enrolled patients with 12 months after PCI and excluded those not providing any prognosis with medical treatment. Patients whose follow-up period was <18 months after PCI were excluded. Patients who died <18 months after PCI were also excluded. We included only those patients who had the same prescriptions between 12 and 18 months after PCI at 3-month intervals. Those who had new clinical events including stroke, acute MI, re-PCI, or bleeding complications within 18 months after PCI were excluded, and only stable patients were included in this analysis. Eventually, the medical records of 4193 patients were assessed according to the antithrombotic regimen (Figure 1). We retrieved inpatient and outpatient prescription records of antithrombotic therapy, including aspirin, clopidogrel, vitamin K antagonist, and non-vitamin K antagonist oral anticoagulants from 1 year after the index PCI. According to the prescription of antithrombotic therapy, patients were categorized as the following 6 groups: no treatment, single antiplatelet therapy (SAPT), OAC monotherapy, dual antiplatelet therapy (DAPT), OAC with SAPT, and OAC with DAPT.

### 2.4. Clinical Outcomes

The primary efficacy endpoint was a composite of major adverse cardiac events (MACE), including all-cause death, MI, and stroke. The safety outcome was bleeding-related events, defined as admission with an endoscopic or endovascular procedure for hemostasis or red blood cell transfusion. Clinical outcomes were followed up from 12 months after PCI. The definitions of clinical outcomes and bleeding-related events are provided in Table S1 in the Supplementary Materials.

### 2.5. Statistical Analysis

Categorical variables are expressed as frequencies (percentages). Differences in categorical variables between groups were assessed using Pearson’s chi-square tests or Fisher’s exact test, as appropriate. Continuous data are expressed as mean ± SD and compared using the one-way ANOVA test. Event-free survival curves as a landmark analysis at 18 months after index PCI were constructed using the Kaplan–Meier method, and statistical differences between the curves were assessed using the log-rank test. Cox proportional hazard models were used to calculate hazard ratios HRs and 95% confidence intervals. Multivariate models were constructed to adjust covariates including age, sex, baseline risk factors, pre-existing cardiovascular disease, and CHA_2_DS_2_-VASc. The propensity score was obtained with the use of logistic regression to adjust for between-group differences in the baseline characteristics of patients. Inverse probability weighting based on propensity scores was then used as the primary tool to adjust for differences between the two treatment groups. A *p* value <0.05 was considered statistically significant. Statistical comparisons were performed using the R software, version 3.3.3 (the R Foundation for Statistical Computing, Vienna, Austria, 4 February 2020).

## 3. Results

### 3.1. Baseline Characteristics

A total of 4193 patients with AF who were stable for 18 months after index PCI were included in the analyses. Out of the total population, 15.1% of patients received no treatment, 20.4% SAPT, 3.3% OAC monotherapy, 37.2% DAPT, 21.0% OAC with SAPT, and 3.0% OAC with DAPT. The baseline characteristics are shown in Table 1. The mean age was 69.8 years, and 68.5% were men. Among the study population, 81.6% received antiplatelet therapy, whereas only 27.3% had OAC-based therapy at 18 months after PCI. The dominant therapy was DAPT (37.2%, *n* = 1560). Only 3.3% of participants had OAC monotherapy (*n* = 138) and another 21.0% (*n* = 879) had OAC with SAPT. Patients receiving OAC monotherapy were significantly older, were more likely to be women, have a history of MI, peripheral artery disease, heart failure, or stroke, and had lower CHA_2_DS_2_-VASc scores than the other groups. Propensity score weighting was performed to compare OAC monotherapy and OAC with SAPT without differences in covariates, and the two treatment groups were well balanced in all variables (Supplementary Table S2).

### 3.2. Clinical Outcomes

**Figure 2:**
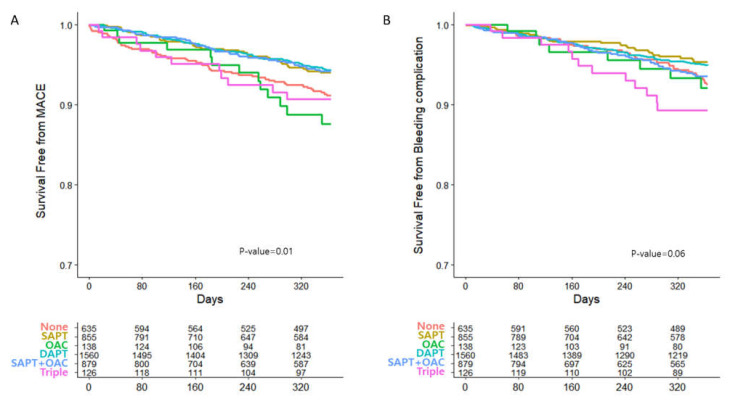
Kaplan–Meier Curves for primary efficacy and safety endpoints in the total study groups. (**A**) The primary efficacy outcome is MACE defined as a composite of all-cause death, myocardial infarction and stroke. (**B**) The safety outcome is bleeding events defined as an admission with an endoscopic or endovascular procedure for hemostasis or red blood cell transfusion. DAPT, dual antiplatelet therapy; MACE, major adverse cardiac events; OAC, oral anticoagulant; SAPT, single antiplatelet therapy.

The outcomes of all patients are shown in Table 2. At the 1-year follow-up from 12 months after PCI, the primary efficacy end point occurred in 242 patients (5.8%), and bleeding-related events occurred in 207 patients (4.9%) from the total study population. The OAC monotherapy group showed the highest MACE (9.42%), followed by the triple-antithrombotic (8.73%) and no treatment groups (8.19%). The OAC with SAPT group showed the lowest MACE (4.78%). Bleeding complications occurred most frequently in the triple-antithrombotic group (9.52%). The Kaplan–Meier curves of each group for MACE showed a significant difference. No medication, OAC alone, and triple-antithrombotic groups showed worse outcomes than the other groups (Figure 2). The probability of bleeding complications among the six groups was not significantly different.

The incidence of MACE was significantly lower among those receiving OAC with SAPT than among those receiving OAC monotherapy (4.78% vs. 9.42%, *p* = 0.017) (Table 2). The incidence of all-cause mortality and MI were similar between patients receiving OAC with SAPT and those receiving OAC monotherapy. However, the incidence of stroke was significantly lower in the OAC with SAPT group than in the OAC monotherapy group, with rates of 1.37% and 4.35%, respectively (*p* = 0.009). Bleeding complication events (5.01% vs. 5.80%, *p* = 0.587) did not differ between the two groups.

In the univariate and multivariate Cox proportional hazards models, the risks resembled those in the main analysis. The risk of MACE was significantly lower in those who were also prescribed an antiplatelet agent than in those who were not, whereas the risk of bleeding was comparable between the OAC with SAPT and OAC monotherapy groups (Table 3).

**Figure 3:**
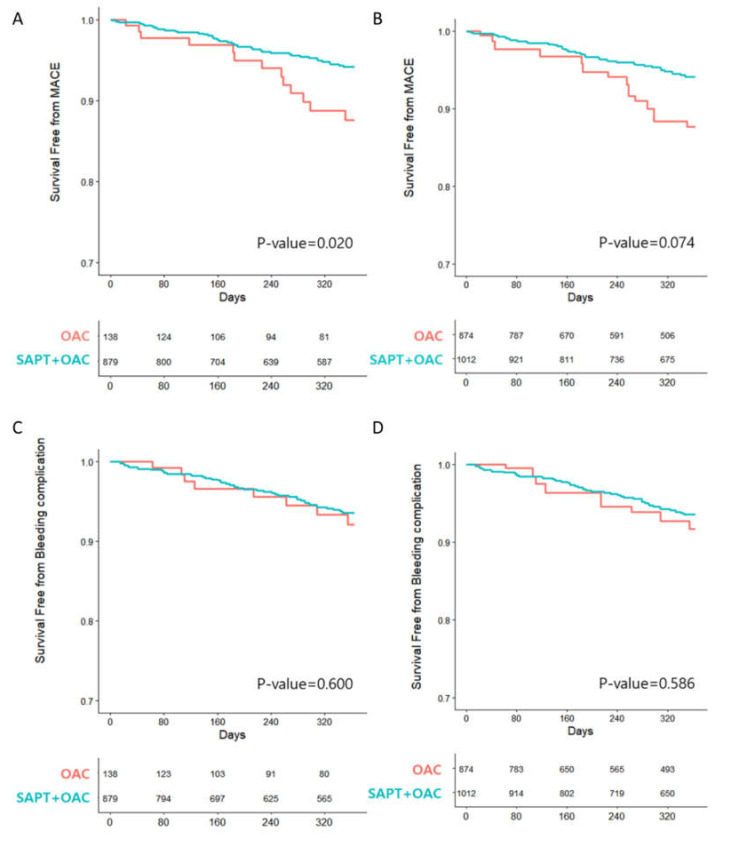
Kaplan–Meier curves of the crude population and inverse probability of treatment weighting (IPTW) population for the clinical outcomes in OAC with SAPT and OAC monotherapy. MACE of (**A**) unadjusted and (**B**) IPTW-adjusted population; bleeding complication of (**C**) unadjusted and (**D**) IPTW-adjusted population. MACE, major adverse cardiac events; OAC, oral anticoagulant; SAPT, single antiplatelet therapy.

Kaplan–Meier curves of the crude population and inverse probability of treatment weighting population of the two groups for the outcomes are displayed in Figure 3. After adjusting for the different baseline characteristics, the probability of MACE and bleeding complications between the two groups were not significantly different, although the OCA with SAPT group showed a trend for a lower incidence of MACE than the group with OAC alone.

## 4. Discussion

In this study, we found that patients with AF received various antithrombotic regimens, and OAC monotherapy was an uncommon treatment beyond 1 year after PCI. Antiplatelet-based therapy was used in 81.6% of the participants. The main finding of this study was that the incidence of MACE was significantly different according to the combinations of antithrombotics, while the incidence of bleeding complications was not. Addition of SAPT to OAC therapy seemed to be associated with a lower risk of MACE than OAC alone, without increasing the incidence of bleeding events. However, the difference in the risk of MACE between patients who received OAC with and without SAPT was altered by statistical adjustment methods, suggesting that there is uncertainty regarding OAC with SAPT being a better choice than OAC alone after 1 year in patients with AF undergoing PCI. These results show a gap between the routine clinical practice and the current guidelines that recommend life-long OAC monotherapy beyond the 1-year time point after PCI. Contemporary guidelines recommend a short period of triple therapy (OAC, aspirin, clopidogrel) followed by a period of dual therapy during the first 12 months. After 12 months of combination therapy, OAC monotherapy is recommended [5,6,7]. However, as reflected in this study, in routine clinical practice, antiplatelet agents are commonly used in combination with OAC beyond 1 year after coronary stenting, mainly because of concerns related to the risk of stent thrombosis [12,13,14]. In a population-based study of 8891 Korean patients between 2009 and 2013, Park et al. observed that 15% of patients with AF received OAC 1 year after PCI [14]. The prescription rate of OAC 1 year after PCI was higher in our study (27.3% vs. 15.2%), which indicated a gradual increase in the adoption of an OAC-based regimen after PCI over time, particularly after the approval of non-vitamin K antagonist oral anticoagulants in 2013. However, the treatment pattern of maintaining prolonged antiplatelet therapy (SAPT or DAPT) was similar in patients on OAC at a year after PCI (88.0% vs. 90.1%).

Another important finding of this study is that a significant proportion (15.1%) of the patients had no antithrombotic therapy and showed poor outcomes in terms of MACE as well as bleeding complications. In this cohort study, it is difficult to determine why these patients did not receive any antithrombotics. Although we excluded patients with any ischemic or bleeding complications within 18 months post-PCI to overcome confounding by indication, there might be a potential selection bias induced by measured and unmeasured confounders. As described in Table 1, these patients had more frequent renal disease. Moreover, we lacked information on labile International Normalized Ratio, alcohol intake, concomitant medication, anthropometric and behavioral factors, or other clinical situations that might affect the antithrombotic regimen. Nevertheless, it is of paramount importance to investigate why the drug was not prescribed and how to improve their prognosis.

The current guidelines are not supported by definite evidence in this phase. Data on the timing of cessation of antiplatelet agents in stented patients requiring long-term OAC are scarce. Previous studies tried to establish a regimen of antithrombotic therapy beyond 1 year in patients with AF who underwent PCI. The OAC-ALONE trial, which is the first randomized trial comparing OAC alone and a combination of OAC and SAPT, did not establish the non-inferiority of OAC alone to combined OAC and SAPT in patients with AF and stable CAD beyond 1 year after stenting, because patient enrollment was prematurely terminated, leading to an underpowered sample size [15]. The OLTAT Registry failed to show the beneficial effect of adding an antiplatelet agent to OAC in patients with AF and stable CAD beyond 1 year after stenting [16]. There were no differences in endpoints in terms of efficacy, but significant bleeding was more evident in patients administered an antiplatelet agent. However, in that study, 70% of patients received warfarin as an OAC, and an all-cause death rate of 30% was extraordinary.

Recently, the AFIRE trials demonstrated that rivaroxaban monotherapy was not inferior to a combination of rivaroxaban and an antiplatelet agent with respect to cardiovascular events and death from any cause, and was superior with respect to major bleeding in patients with AF and stable CAD [17]. In the AFIRE study, 70.6% of patients received PCI, among whom 23% received bare metal stent. Meanwhile, 11.4% had previously undergone CABG and 19% patients received only medical therapy. In contrast, 100% of patients in our study received stents. In addition, aspirin was chosen as the antiplatelet agent in 70% of patients, while P2Y12 inhibitors were used in 71.2% of the OAC with SAPT group in our study. The patients in the AFIRE study had lesser previous MI and stroke than those in our study. In clinical practice, we often encounter patients with higher risk and more advanced atherosclerotic diseases. Therefore, we should prudently apply the result of a single randomized controlled trial in clinical practice. Our study found that OAC with SAPT beyond 1 year after PCI reduced ischemic events without increasing bleeding complications in patients with AF. High bleeding risk factors should be appropriately identified and managed in clinical practice. In terms of discriminating bleeding risk, the Academic Research Consortium High Bleeding Risk framework has been validated and showed an overall good performance in patients undergoing PCI receiving OAC therapy [18].

Limitations and biases were inherent to the observational nature of the analysis, which included an actual clinical, unselected cohort of patients with AF who underwent PCI. Information regarding medication compliance in patients was unavailable in our study, which may affect the actual proportion of patients receiving different antithrombotic regimens, although we included only those who had the same prescriptions between 12 and 18 months after PCI at 3-month intervals to minimize misclassification bias. Survival treatment selection bias cannot be excluded because patients who were stable with a certain antithrombotic regimen for 18 months had a higher probability of receiving the same treatment, which could yield a positive association. IPTW was used to adjust for differences between groups, but confounding factors could still remain. However, the large sample size in this study reduces the potential uncertainty and bias. There is a limitation in that the OAC monotherapy group included a relatively small number of patients. Because the presence of major bleeding was determined using claims data, the definition used for major bleeding is not standardized. Finally, information regarding the complexity of the coronary lesions and the PCI procedure, which may affect the selection of the antithrombotic strategy, were not included in the analysis.

## 5. Conclusions

In this study, antiplatelet-based therapy is the mainstream antithrombotic regimen, and OAC monotherapy is a minor treatment in AF patients beyond 1 year after PCI. Addition of SAPT to OAC therapy seemed to be associated with a lower risk of MACE than OAC alone, without increasing the rate of bleeding events. However, it is uncertain whether OAC with SAPT would be a better choice. Therefore, further randomized controlled trials are needed to conclusively determine the optimal antithrombotic therapy regimen beyond 1 year after PCI in patients with AF.

## Figures and Tables

**Table 1:** Baseline characteristics of the total study population based on combinations of anticoagulants and antiplatelet agents.

Variables	Total	No Treatment	SAPT	OAC Monotherapy	DAPT	OAC with SAPT	OAC with DAPT	*p*-Value
	(*n* = 4193)	(*n* = 635)	(*n* = 855)	(*n* = 138)	(*n* = 1560)	(*n* = 879)	(*n* = 126)	
Age, years	69.8 ± 10.6	70.8 ± 10.8	69.0 ± 10.9	72.3 ± 8.4	68.9 ± 11.1	71.0 ± 9.2	68.5 ± 10.2	<0.001
Male	2874 (68.5)	443 (69.7)	560 (65.5)	77 (55.8)	1090 (69.8)	611 (69.5)	93 (73.8)	0.003
Baseline risk factors								
Hypertension	3595 (85.7)	541 (85.2)	720 (84.2)	125 (90.5)	1302 (83.4)	797 (90.6)	110 (87.3)	<0.001
Diabetes mellitus	1494 (35.6)	243 (38.2)	280 (32.7)	49 (35.5)	556 (35.6)	312 (35.4)	54 (42.8)	0.162
Renal disease	398 (9.4)	121 (19.0)	65 (7.6)	12 (8.7)	121 (7.7)	70 (7.9)	9 (7.1)	<0.001
Liver disease	108 (2.5)	20 (3.1)	19 (2.2)	6 (4.3)	43 (2.7)	17 (1.9)	3 (2.3)	0.460
Dyslipidemia	4060 (96.8)	621 (97.8)	829 (96.9)	133 (96.3)	1506 (96.5)	848 (96.4)	123 (97.6)	0.682
Pre-existing cardiovascular disease								
Ischemic heart disease	4186 (99.8)	634 (99.8)	854 (99.8)	137 (99.2)	1559 (99.9)	877 (99.7)	125 (99.2)	0.231
Prior myocardial infarction	1810 (43.1)	287 (45.2)	364 (42.5)	45 (32.6)	695 (44.5)	358 (40.7)	61 (48.4)	0.032
Peripheral artery disease	1134 (27.0)	207 (32.6)	216 (25.2)	41 (29.7)	414 (26.5)	227 (25.8)	29 (23.0)	0.018
Prior stroke	1498 (35.7)	260 (40.9)	247 (28.8)	67 (48.5)	515 (33.0)	360 (40.9)	49 (38.8)	<0.001
Heart failure	2396 (57.1)	384 (60.4)	427 (49.9)	92 (66.6)	854 (54.7)	562 (63.9)	77 (61.1)	<0.001
Atrial fibrillation	3479 (82.9)	539 (84.8)	692 (80.9)	120 (86.9)	1227 (78.6)	795 (90.4)	106 (84.1)	<0.001
CHA_2_DS_2_-VASc score	4.90 (1.9)	5.10 (1.8)	4.63 (2.0)	5.62 (1.8)	4.72 (2.0)	5.21 (1.7)	4.95 (1.9)	<0.001
Medications use								
Aspirin	2395 (57.1)	0 (0)	462 (54.0)	0 (0)	1560 (100)	247 (28.1)	126 (100)	
P2Y_12_ inhibitors	2711 (64.6)	0 (0)	393 (45.9)	0 (0)	1560 (100)	632 (71.9)	126 (100)	
Warfarin	507 (12.0)	0 (0)	0 (0)	42 (30.4)	0 (0)	372 (42.3)	93 (73.8)	
OAC	636 (15.1)	0 (0)	0 (0)	96 (69.5)	0 (0)	507 (57.6)	33 (26.1)	

DAPT, dual antiplatelet therapy; OAC, oral anticoagulant; SAPT, single antiplatelet therapy. Age and CHA_2_DS_2_-VASc scores were analyzed by ANOVA test. The others were analyzed by chi-square test.

**Table 2:** 1-year clinical outcomes according to combinations of anticoagulants and antiplatelet agents.

(**A**)								
**Outcomes**	**Total**	**No Treatment**	**SAPT**	**OAC Monotherapy**	**DAPT**	**OAC with SAPT**	**OAC with DAPT**	***p*-Value**
	**(*n* = 4193)**	**(*n* = 635)**	**(*n* = 855)**	**(*n* = 138)**	**(*n* = 1560)**	**(*n* = 879)**	**(*n* = 126)**	
Primary efficacy end point								
MACE	242 (5.7)	52 (8.1)	43 (5.0)	13 (9.4)	81 (5.1)	42 (4.7)	11 (8.7)	0.008
All-cause of death	172 (4.1)	40 (6.3)	31 (3.6)	8 (5.8)	55 (3.5)	32 (3.6)	6 (4.7)	0.047
MI	23 (0.5)	4 (0.6)	6 (0.7)	1 (0.7)	6 (0.3)	5 (0.5)	1 (0.7)	0.92
Stroke	77 (1.8)	11 (1.7)	14 (1.6)	6 (4.3)	29 (1.8)	12 (1.3)	5 (3.9)	0.097
Primary safety end point								
Bleeding complication	207 (4.9)	41 (6.4)	32 (3.7)	8 (5.8)	70 (4.4)	44 (5.0)	12 (9.5)	0.031
(**B**)								
**Outcomes**		**OAC Monotherapy**		**OAC with SAPT**		***p*-Value**		
		**(*n* = 138)**		**(*n* = 879)**				
Primary efficacy end point								
MACE		13 (9.4)		42 (4.7)		0.017		
All-cause of death		8 (5.8)		32 (3.6)		0.175		
MI		1 (0.7)		5 (0.5)		0.798		
Stroke		6 (4.3)		12 (1.3)		0.009		
Primary safety end point								
Bleeding complication		8 (5.8)		44 (5.0)		0.587		

DAPT, dual antiplatelet therapy; MACE, major adverse cardiac events; MI, myocardial infarction; OAC, oral anticoagulant; SAPT, single antiplatelet therapy.

**Table 3:** Comparison of univariate and multivariate predictors of MACE and bleeding complications between OAC with SAPT and OAC monotherapy.

Outcomes	Hazard Ratio	95% CI	*p*-Value
Univariate			
MACE	0.48	0.26–0.89	0.019
Bleeding complications	0.83	0.39–1.76	0.630
Adjusted by age, sex			
MACE	0.48	0.25–0.89	0.020
Bleeding complications	0.84	0.39–1.79	0.651
Adjusted by CHA_2_DS_2_-VASc			
MACE	0.52	0.28–0.97	0.040
Bleeding complications	0.87	0.41–1.86	0.726
Adjusted by all covariates			
MACE	0.52	0.28–0.98	0.043
Bleeding complications	0.86	0.40–1.84	0.697

CI, confidence interval; MACE, major adverse cardiac events.

## Data Availability

Publicly available datasets were analyzed in this study. This data can be found here after completion of necessary processes: https://nhiss.nhis.or.kr/bd/ay/bdaya001iv.do.

## Supplementary Materials

The following are available online at https://www.mdpi.com/article/10.3390/jcm10153370/s1, Table S1: Definitions of co-morbidities and clinical outcomes. Table S2: Baseline characteristics of patients who were treated with OAC monotherapy versus OAC with SAPT in total study and weighted study populations.

## References

[1] Angiolillo D.J., Goodman S.G., Bhatt D.L., Eikelboom J.W., Price M.J., Moliterno D.J., Cannon C.P., Tanguay J.F., Granger C.B., Mauri L. (2016). Antithrombotic Therapy in Patients with Atrial Fibrillation Undergoing Percutaneous Coronary Intervention: A North American Perspective—2016 Update. Circ. Cardiovasc. Interv..

[2] Kralev S., Schneider K., Lang S., Suselbeck T., Borggrefe M. (2011). Incidence and severity of coronary artery disease in patients with atrial fibrillation undergoing first-time coronary angiography. PLoS ONE.

[3] Michniewicz E., Mlodawska E., Lopatowska P., Tomaszuk-Kazberuk A., Malyszko J. (2018). Patients with atrial fibrillation and coronary artery disease—Double trouble. Adv. Med. Sci..

[4] Lip G., Freedman B., De Caterina R., Potpara T.S. (2017). Stroke prevention in atrial fibrillation: Past, present and future. Comparing the guidelines and practical decision-making. Thromb. Haemost..

[5] Valgimigli M., Bueno H., Byrne R.A., Collet J.P., Costa F., Jeppsson A., Juni P., Kastrati A., Kolh P., Mauri L. (2018). 2017 ESC focused update on dual antiplatelet therapy in coronary artery disease developed in collaboration with EACTS: The Task Force for dual antiplatelet therapy in coronary artery disease of the European Society of Cardiology (ESC) and of the European Association for Cardio-Thoracic Surgery (EACTS). Eur. Heart J..

[6] Hindricks G., Potpara T., Nikolaos D., Arbelo E., Bax J.J., Blomström-Lundqvist C., Boriani G., Castella M., Dan G., Dilaveris P.E. (2021). 2020 ESC Guidelines for the diagnosis and management of atrial fibrillation developed in collaboration with the European Association for Cardio-Thoracic Surgery (EACTS): The Task Force for the diagnosis and management of atrial fibrillation of the European Society of Cardiology (ESC) Developed with the special contribution of the European Heart Rhythm Association (EHRA) of the ESC. Eur. Heart J..

[7] Angiolillo D.J., Bhatt D.L., Cannon C.P., Eikelboom J.W., Gibson C.M., Goodman S.G., Granger C.B., Holmes D.R., Lopes R.D., Mehran R. (2021). Antithrombotic Therapy in Patients with Atrial Fibrillation Treated with Oral Anticoagulation Undergoing Percutaneous Coronary Intervention: A North American Perspective: 2021 Update. Circulation.

[8] Lam D.H., Bell S.M., Hira R.S. (2018). Concomitant Use of Antiplatelets and Anticoagulants in Patients with Coronary Heart Disease and Atrial Fibrillation: What Do Recent Clinical Trials Teach Us?. Curr. Atheroscler. Rep..

[9] Dans A.L., Connolly S.J., Wallentin L., Yang S., Nakamya J., Brueckmann M., Ezekowitz M., Oldgren J., Eikelboom J.W., Reilly P.A. (2013). Concomitant use of antiplatelet therapy with dabigatran or warfarin in the Randomized Evaluation of Long-Term Anticoagulation Therapy (RE-LY) trial. Circulation.

[10] Alexander J.H., Wojdyla D., Vora A.N., Thomas L., Granger C.B., Goodman S.G., Aronson R., Windecker S., Mehran R., Lopes R.D. (2020). Risk/Benefit Tradeoff of Antithrombotic Therapy in Patients with Atrial Fibrillation Early and Late after an Acute Coronary Syndrome or Percutaneous Coronary Intervention. Circulation.

[11] Andò G., Costa F. (2020). Double or triple antithrombotic therapy after coronary stenting and atrial fibrillation: A systematic review and meta-analysis of randomized clinical trials. Int. J. Cardiol..

[12] Ancedy Y., Lecoq C., Saint Etienne C., Ivanes F., Angoulvant D., Babuty D., Lip G.Y., Fauchier L. (2016). Antithrombotic management in patients with atrial fibrillation undergoing coronary stent implantation: What is the impact of guideline adherence?. Int. J. Cardiol..

[13] Ono F., Tanaka S., Nakao Y.M., Kawakami K. (2018). Utilization of Anticoagulant and Antiplatelet Agents Among Patients with Atrial Fibrillation Undergoing Percutaneous Coronary Intervention- Retrospective Cohort Study Using a Nationwide Claims Database in Japan. Circ. J..

[14] Park J., Choi E.K., Han K.D., Choi Y.J., Lee S.R., Cha M.J., Kang J., Park K.W., Oh S., Lip G.Y.H. (2019). Antithrombotic Therapy in Patients with Atrial Fibrillation after Percutaneous Coronary Intervention During 2-Year Follow-Up, from a Nationwide Population Study. Am. J. Circ..

[15] Matsumura-Nakano Y., Shizuta S., Komasa A., Morimoto T., Masuda H., Shiomi H., Goto K., Nakai K., Ogawa H., Kobori A. (2019). Open-Label Randomized Trial Comparing Oral Anticoagulation with and without Single Antiplatelet Therapy in Patients with Atrial Fibrillation and Stable Coronary Artery Disease beyond 1 Year after Coronary Stent Implantation. Circulation.

[16] Fischer Q., Georges J.L., Feuvre C.L., Sharma A., Hammoudi N., Berman E., Cohen S., Jolivet I., Silvain J., Helft G. (2018). Optimal long-term antithrombotic treatment of patients with stable coronary artery disease and atrial fibrillation: “OLTAT registry”. Int. J. Cardiol..

[17] Yasuda S., Kaikita K., Akao M., Ako J., Matoba T., Nakamura M., Miyauchi K., Hagiwara N., Kimura K., Hirayama A. (2019). Antithrombotic Therapy for Atrial Fibrillation with Stable Coronary Disease. N. Eng. J. Med..

[18] Gragnano F., Spirito A., Corpataux N., Vaisnora L., Galea R., Gargiulo G., Siontis G.C.M., Praz F., Lanz J., Billinger M. (2021). Impact of Clinical Presentation on Bleeding Risk after Percutaneous Coronary Intervention and Implications for the ARC-HBR Definition. EuroIntervention.

