# Supplementary material for: Prognosis of Atrial Fibrillation Patients Undergoing PCI According to Anticoagulants and Antiplatelet Agents
Source: J Clin Med. 2021 Jul 29;10(15):3370. doi: 10.3390/jcm10153370 (PMC8348599; doi:10.3390/jcm10153370)
Supplement: Supplementary file 1 [file jcm-10-03370-s001.zip › jcm-1305867-supplementary.pdf]

Journal of Clinical Medicine

Article

Supplementary Material: Prognosis of Atrial Fibrillation Patients Undergoing PCI According to Anticoagulants and Antiplatelet Agents

Gwang-Seok Yoon, Sun-Hwa Kim, Si-Hyuck Kang, Chang-Hwan Yoon, Young-Seok Cho, Tae-Jin Youn and In-Ho Chae

Table S1. Definitions of co-morbidities and clinical outcomes.

Diagnosis	Definition(ICD-10Codes)		DiagnosticDefinition
Hypertension	I10	Essential(primary)hypertension	Admission≥1oroutpatientdepartment≥2withmedication
	I11	Hypertensiveheartdisease	
	I12	Hypertensiverenaldisease	
	I13	Hypertensiveheartandrenaldisease	
	I15	Secondaryhypertension	
Diabetesmellitus	E11	Type2diabetesmellitus	Admission≥1oroutpatientdepartment≥2withmedication
	E12	Malnutrition-relateddiabetesmellitus	
	E13	Otherspecifieddiabetesmellitus	
	E14	Unspecifieddiabetesmellitus	
Dyslipidemia	E78	Disordersoflipoproteinmetabolismandotherlipidemias	Admission≥1oroutpatientdepartment≥2
Ischemicheartdisease	I20	Anginapectoris	Admissionoroutpatientdepartment≥1
	I21	Acutemyocardialinfarction	
	I22	Subsequentmyocardialinfarction	
	I23	Certaincurrentcomplicationsfollowingacutemyocardialinfarction	
	I24	Otheracuteischemicheartdiseases	
	I25	Chronicischemicheartdisease	
Myocardialinfarction	I21	Acutemyocardialinfarction	1)BeforeindexPCIdate:admission≥1oroutpatientdepartment≥2
	I23	Certaincurrentcomplicationsfollowingacutemyocardialinfarction	2)AfterindexPCIdate:admission≥1
Peripheralarterydisease	I70	Atherosclerosis	Admissionoroutpatientdepartment≥1
	I73	Otherperipheralvasculardiseases	
Stroke	I60	Subarachnoidhemorrhage	Admission≥1andbrainimaging(CTorMRI)≥1
	I61	Intracerebralhemorrhage	
	I62	Othernontraumaticintracranialhemorrhage	
	I63	Cerebralinfarction	
	I64	Stroke,notspecifiedashemorrhageorinfarction	
Heartfailure	I50	Heartfailure	Admissionoroutpatientdepartment≥1
Atrialfibrillation	I480	Paroxysmalatrialfibrillation	Admissionoroutpatientdepartment≥1
	I481	Persistentatrialfibrillation	
	I482	Chronicatrialfibrillation	
	I483	Typicalatrialflutter	
	I484	Atypicalatrialflutter	
	I489	Unspecifiedatrialfibrillationandatrialflutter	
Majorbleeding		Intracranialbleeding	ICH,admission≥1orRBCtransfusion≥1
		Gastrointestinalbleeding	Admissionandendoscopichemostasisprocedure
		Otherbleeding	Admissionandendovascularembolizationforhemostasisorbloodtransfusion

CT, computer tomography; ICD, international classification of disease; ICH, intracerebral hemorrhage; MRI, magnetic resonance imaging; PCI, percutaneous coronary intervention; RBC, red blood cell.

Table S2. Baseline characteristics of patients who were treated with OAC monotherapy versus OAC with SAPT in total study and weighted study populations.

Variables	Overall Cohort				IPTW Cohort				
	Total	OAC Monotherapy	OAC with SAPT	*p* Value	Total	OAC Monotherapy	OAC with SAPT	*p*-Value	Standardized Difference
Age, years	71.2 ± 9.1	72.3 ± 8.4	71.0 ± 9.2	<.001	71.5 ± 8.8	71.9 ± 8.3	71.2 ± 9.1	0.403	0.078
Male, n (%)	688 (67.6)	77 (55.8)	611 (69.5)	0.002	1264.8 (67.0)	576 (65.9)	688 (68.0)	0.634	0.044
Baseline risk factors									
Hypertension	922 (90.6)	125 (90.5)	797 (90.6)	>.999	1709 (90.6)	791 (90.5)	918 (90.7)	0.934	0.088
Diabetes mellitus	361 (35.5)	49 (35.5)	312 (35.4)	>.999	633(35.2)	302 (34.5)	361 (35.7)	0.797	0.025
Renal disease	82 (8.0)	12 (8.7)	70 (7.9)	0.900	156 (35.7)	75 (8.6)	81 (8.0)	0.812	0.023
Dyslipidemia	981 (96.4)	133 (96.3)	848 (96.4)	>.999	1827 (96.9)	851 (97.4)	976 (96.4)	0.530	0.050
Ischemic heart disease	1014 (99.7)	137 (99.2)	877 (99.7)	0.875	1875 (99.4)	866 (99.0)	1009 (99.7)	0.239	0.138
Prior myocardial infarction	403 (39.6)	45 (32.6)	358 (40.7)	0.086	706 (67.4)	302 (34.6)	404 (39.9)	0.271	0.109
Peripheral artery disease	268 (26.3)	41 (29.7)	227 (25.8)	0.390	493 (26.1)	228 (26.1)	265 (26.1)	0.988	0.001
Prior stroke	427 (41.9)	67 (48.5)	360 (40.9)	0.112	812 (43.1)	391 (44.8)	241 (41.6)	0.502	0.065
Heart failure	654 (64.3)	92 (66.6)	562 (63.9)	0.598	1211 (64.2)	562 (64.3)	649 (64.2)	0.982	0.002
Liver disease	23 (2.2)	6 (4.3)	17 (1.9)	0.143	42 (2.2)	21 (2.4)	21 (2.1)	0.783	0.021
Atrial fibrillation	915 (89.9)	120 (86.9)	795 (90.4)	0.265	1705 (90.4)	791 (90.5)	913 (90.2)	0.906	0.010
CHA_2DS_2-VASc score	5.2 (1.8)	5.6 (1.8)	5.2 (1.7)	<.001	5.3 (1.8)	5.3 (1.8)	5.2 (1.8)	0.983	0.003

IPTW, Inverse Probability Treatment Weighting; OAC, oral anticoagulant; SAPT, single antiplatelet therapy.
